# Atorvastatin-loaded nanostructured lipid carriers (NLCs): strategy to overcome oral delivery drawbacks

**DOI:** 10.1080/10717544.2017.1337823

**Published:** 2017-06-15

**Authors:** Mohammed Elmowafy, Hany M. Ibrahim, Mohammed A. Ahmed, Khaled Shalaby, Ayman Salama, Hossam Hefesha

**Affiliations:** a Department of Pharmaceutics and Industrial Pharmacy, Faculty of Pharmacy, Al-Azhar University, Cairo, Egypt;; b Department of Pharmaceutics and Industrial Pharmacy, Faculty of Pharmacy, Cairo University, Cairo, Egypt

**Keywords:** Atorvastatin, nanostructured lipid carriers (NLCs), oral delivery, bioavailability, pharmacodynamic effects

## Abstract

Atorvastatin (AT) is a widely used lipid-regulating drug to reduce cholesterol and triglycerides. Its poor aqueous solubility and hepatic metabolism require development of drug delivery systems able to improve its solubility and bypass hepatic effect. For this purpose, atorvastatin nanostructured lipid carriers (AT-NLCs) were prepared and characterized. AT-NLCs were prepared by emulsification using high-speed homogenization followed by ultrasonication. The prepared NLCs showed particle size between 162.5 ± 12 and 865.55 ± 28 nm while zeta potential values varied between −34 ± 0.29 and −23 ± 0.36 mV. They also showed high encapsulation efficiency (>87%) and amorphous state of the drug in lipid matrix. Pharmacokinetic parameters of optimized formulation (NLC-1; composed of 2% Gelucire^®^ 43/01, 8% Capryol^®^ PGMC, 2% Pluronic^®^F68 and 0.5% lecithin) revealed 3.6- and 2.1-fold increase in bioavailability as compared to atorvastatin suspension and commercial product (Lipitor^®^), respectively. Administration of NLC-1 led to significant reduction (*p* < .05) in the rats’ serum levels of total cholesterol (TC), triglyceride (TG), low-density lipoprotein (LDL) and significant increase in high-density lipoprotein (HDL). This improvement was confirmed histologically by minimizing the associated hepatic steatosis. These investigations demonstrated the superiority of NLCs for improvement of oral bioavailability and *in vivo* performance of AT.

## 1. Introduction

Atorvastatin (AT) is 3-hydroxy-3-methylglutaryl coenzyme A (HMG-CoA) reductase competitive inhibitor, the enzyme which catalyzes the conversion of HMG-CoA to mevalonate, an early and a rate limiting step in cholesterol biosynthesis. AT is a member of the class of drugs known as statins, used to decrease blood cholesterol level. It has been also used in the primary and secondary prevention of cardiovascular events (Kim et al., 2008).

According to Biopharmaceutical Classification System (BCS), AT belongs to class II with a very good intestinal permeability and low aqueous solubility (0.1 mg/ml). However, it has low oral bioavailability (12%) as a result of low aqueous solubility, crystalline nature, rapid presystemic clearance in the gut wall and hepatic first-pass metabolism (Lau et al., 2006). Poor performance of AT leads to administration in high doses possibly leading to liver abnormalities, rhabdomyolysis, arthralgia and kidney failure (Choudhary et al., 2012). So, improvement of solubility and hence oral bioavailability of crystalline AT can be achieved through developing a method that reduces the particle size and maintains the drug in an amorphous state (Anwar et al., 2011).

Lipid-based drug delivery systems are promising drug carriers due to their ability to improve solubility of poorly water-soluble and/or lipophilic drugs which eventually enhance the oral bioavailability (O'Driscoll & Griffin, 2008). The first generation of lipidic nanoparticles, the so-called solid lipid nanoparticles (SLNs), is composed of an aqueous dispersion of nanoparticles with a solid lipid matrix that is stabilized by one or more of surfactant layer. However, SLNs presented some drawbacks, such as limited drug loading capacity and potential tendency for drug expulsion during storage. Therefore, there is necessity to create second generation of lipidic nanoparticles, the nanostructured lipid carriers (NLCs). In contrast to SLNs, dispersions of NLCs are formed of a blend of solid lipid with liquid lipid, which provide a higher payload and prevent drug expulsion during storage (Saupe et al., 2005; Muller et al., 2007). Higher drug loading is attributed to the differences in the chemical structure between liquid and solid lipids, which result in distortion of a perfect crystal and accommodation of drug in molecular form or in amorphous clusters (Muller et al., 2007). Moreover, NLC formulations have the advantages of prolonged drug release, biocompatibility and easy of scaling-up its production (Wang & Xia, 2014). The *in vivo* digestion (by lipases and co-lipases) of NLCs, once reaching the small intestine, converts them into mixed micelles that are ready for drug absorption (Yu et al., 2016). The lymphatic route or payer's patches may have a role in improving oral absorption of drugs encapsulated in NLCs (Charman, 2000; Porter, 2001).

The objective of the present study was to explore the feasibility to encapsulate AT into NLCs to improve dissolution rate and eventually oral bioavailability. Gelucire^®^ 43/01, glycerylmonostearate (GMS) and Compritol^®^ 888 ATO were chosen as solid lipids while Capryol^®^ PGMC was selected as liquid lipid. Pluronic^®^F68, Tween^®^ 80 and their binary mixture (1:1) were used as hydrophilic surfactants, while lecithin was selected as lipophilic emulsifier. NLCs were optimized for physicochemical properties, and pharmacokinetic and pharmacodynamic efficacy in rats was examined. Stability study of AT-NLCs at 2–8 °C and 25 °C for 3 months was evaluated.

## 2. Materials and methods

### 2.1. Materials

Gelucire^®^ 43/01 (mixtures of mono-, di- and triglycerides with PEG esters of fatty acids), glyceryl monostearate (GMS), Compritol^®^ 888 ATO (glycerol dibehenate/behenate) and Capryol^®^ PGMC (propylene glycol monocaprylate type I) were kindly gifted by Gattefosse (St Priest, Cedex, France). Pluronic^®^F68 (polyoxyethylene-polyoxypropylene (150:29) block copolymer, poloxamer 188) was purchased from Sigma-Aldrich, Inc. (St. Louis, MO). AT (atorvastatin) is kindly gifted by Al-Arabiya Pharmaceutical Company (Cairo, Egypt). Lecithin (70% phosphatidylcholine) was kindly gifted by Lipoid (Ludwigshafen, Germany). Tween^®^ 80 (polyoxyethylen-80-sorbitanmonooleat, polysorbate 80) was purchased from ADWIC Chemicals Co. (Cairo, Egypt). All the reagents were of analytical grade and used without further purification.

### 2.2. Preparation of AT-loaded NLCs

The AT-loaded NLCs were prepared by emulsification using high-speed homogenization followed by ultrasonication. The lipid and aqueous phases were prepared separately. Lipid phase consisted of solid lipid (Gelucire^®^ 43/01, GMS or Compritol^®^ 888 ATO; 2%), liquid lipid (Capryol^®^ PGMC; 8%) and 0.5% lecithin as the lipophilic emulsifier, while the aqueous phase consisted of hydrophilic emulsifier (Pluronic^®^F68, Tween^®^ 80 or their binary mixture (1:1); 2%) dissolved in distilled water. AT was dissolved in Capryol^®^ PGMC and then mixed with other lipid phase components. All components of lipid phase were heated separately to 10 °C above solid lipid transition temperatures (Gelucire^®^ 43/01, 43–46 °C; GMS, 58–60 °C and Compritol^®^ 888 ATO, 68–75 °C) for 10 min before mixing. The aqueous phase was added dropwise to the molten lipid phase and mixed using a high-speed homogenizer (Janke & Kunkel, GmbH, Staufen, Germany) at 10,000 rpm for 10 min. The mixture was further treated using a probe-type sonicator (ultrasonic processor, GE130, probe CV18, Newtown, CT) for 15 min. The resultant emulsions were cooled at room temperature. An overview of different formulations prepared by changing type of solid lipids and surfactants is outlined in Table 1.

**Table 1.** Composition of atorvastatin-loaded nanostructured lipid carriers.

	AT-NLCs composition^a^		Physicochemical properties			
Run	Solid lipid (2%, w/w)	Aqueous surfactant (2%, w/w)	Mean particle size ± SD (nm)	Zeta potential ± SD (mV)	Entrapment efficiency ± SD (%)	Polydispersity index (PDI)
NLC-1	Gelucire^®^ 43/01	Pluronic^®^ F68	162.5 ± 12	−34 ± 0.29	90.1 ± 6.5	0.295
NLC-2	Gelucire^®^ 43/01	Tween^®^ 80	199.14 ± 22	−31 ± 0.50	91.3 ± 5.9	0.434
NLC-3	Gelucire^®^ 43/01	Pluronic^®^ F68–Tween^®^ 80 (1:1)	168.54 ± 14	−31 ± 0.25	87.2 ± 7.5	0.261
NLC-4	GMS	Pluronic^®^ F68	184.98 ± 21	−29 ± 2.8	95.3 ± 5.1	0.276
NLC-5	GMS	Tween^®^ 80	234 ± 6.8	−28 ± 2.9	92.2 ± 6.2	0.451
NLC-6	GMS	Pluronic^®^ F68–Tween^®^ 80 (1:1)	254.4 ± 32.1	−29 ± 2.25	87 ± 4	0.571
NLC-7	Compritol^®^ 888 ATO	Pluronic^®^ F68	865.55 ± 28	−28 ± 0.85	94.6 ± 2.6	0.732
NLC-8	Compritol^®^ 888 ATO	Tween^®^ 80	224.7 ± 7.5	−23 ± 0.36	76 ± 12.4	0.62
NLC-9	Compritol^®^ 888 ATO	Pluronic^®^ F68–Tween^®^ 80 (1:1)	449.4 ± 3	−24 ± 0.54	96.6 ± 7.1	0.691

^a^ Each formulation contains 8%, w/w Capryol^®^ PGMC and 0.5%, w/w lecithin.

### 2.3. Drug encapsulation efficiency

Drug encapsulation efficiency (EE) was determined through indirect method where an aliquot (2 ml) of AT-loaded NLCs was centrifuged at 100,000 g for 2 h at 4 °C using a Beckman Optima™ Ultracentrifuge (Optima™ XL, Indianapolis, IN). The proportion of unencapsulated AT in the clear supernatant fluid was measured spectrophotometrically (Shimadzu, the model UV-1800 PC, Kyoto, Japan) at 246 nm against blank. Calibration curve for the validated UV assay of AT was performed on six solutions in the concentration ranges of 2–20 μg/ml. Correlation coefficient was >0.999. Each point represents the mean of three measurements and standard deviation (±SD) was calculated. The encapsulation efficiency of AT was then calculated according to the following equation:

(1) $$EE\%=\left(Da-Df/Da\right)\times 100,$$

where EE%= the percentage of encapsulation efficiency, Da = the amount of added drug during preparation of NLCs and Df = the amount of free drug in the clear supernatant fluid after centrifugation.

### 2.4. Particle size and zeta potential

The mean particle size, polydispersity index (PDI) and zeta potential of the AT-loaded NLC formulations were determined using Malvern^®^ Zetasizer Nano ZS90 (Malvern^®^ Instruments Limited, Worcestershire, UK). All the measurements were made in triplicate after dilution (1:200) with distilled water at room temperature using 90° scattering angle.

### 2.5. Morphology of AT-NLCs

The morphology of the AT-NLCs was observed by transmission electron microscopy (TEM) (JEM-1011; JEOL, Tokyo, Japan) at 60 Kv. Samples were dispersed in distilled water using bath sonicator (Soniclean 160HT, Soniclean Pty. Ltd., Tokyo, Japan) for five minutes and dropped on a carbon-coated copper grid, forming a thin liquid film. The film on the grid was allowed to dry at room temperature and then photographed by TEM.

### 2.6. Thermal analysis

Thermal analysis experiments were performed using differential scanning calorimeter (DSC) (model TA-50 WSI, Shimadzu, Tokyo, Japan). Pure samples of AT and Gelucire^®^ 43/01 were directly subjected to analysis while the selected formulation (NLC-1) was lyophilized (Telstar Cryodos, Terrassa, Spain) using mannitol as cryoprotectant. One milligram of individual component and lyophilized NLC-1 was placed in aluminum pans sealed with a lid along with the standard reference aluminum. Thermograms were recorded between 25 °C and 400 °C at a scan rate of 10 °C/min in the presence of nitrogen at flow rate of 30 ml/min to obtain the endothermic peaks.

### 2.7. In vitro release studies

*In vitro* release tests were performed for pure AT, and selected formulation (NLC-1) *via* reverse dialysis technique using dialysis membrane with a molecular weight cutoff (MWCO) of 12 to 14 kDa (Spectrum Laboratories Inc., Rancho Dominguez, CA). Dialysis membrane was washed before use with distilled water to remove excess glycerin and then soaked overnight in the release medium. Five milligrams of pure AT and equivalent volume of the developed NLC-1 dispersion was suspended in the dissolution vessel of USP apparatus II (Erweka TD6R, Heusenstamm, Germany) containing 500 ml of the release medium (phosphate buffer pH 6.8) at 100 ± 2 rpm and 37 ± 0.5 °C. Numerous dialysis bags containing small volume of the release medium were previously filled and equilibrated with the release medium for a few hours prior to the experiments. At predetermined time intervals, one dialysis bag was withdrawn from the stirred release medium and replaced with equal volume of the fresh medium to maintain a sink condition. AT contents of the dialysis bag were determined spectrophotometrically (Shimadzu, the model UV-1800 PC, Kyoto, Japan) at 246 nm. All measurements were run in triplicate against blank.

The kinetic parameters for the *in vitro* release data were determined using a special computer program in order to find the most relevant order or the model of AT release. The study included zero-order release kinetic model, first-order release kinetic model Higuchi diffusion model (Higuchi, 1963) and Hixson–Crowell model. The highest correlation coefficient (*r*) value represents the actual mode of the release (Guinedi et al., 2005).

### 2.8. Stability of AT-NLCs

It is important to evaluate stability of the developed NLC formulations. NLC-1 was stored in amber glass bottle covered with aluminum foil at 2–8 and 25 °C. Samples were withdrawn monthly for three months and analyzed with respect to particle size, PDI, zeta potential and entrapment efficiency.

### 2.9. In vivo studies

This study was performed to explore the bioavailability and pharmacodynamic efficacy of AT-loaded NLCs as compared to AT suspension and commercial tablet (Lipitor^®^ 20 mg). Healthy three-month male albino rats weighing 200–250 g were purchased from the animal house colony of the National Research Center (Dokki, Giza, Egypt). Animal studies were carried out in accordance with procedures approved by the Animal Care and Use Committee. Rats were acclimatized in the animal house facilities for one week prior to the experiments under constant environmental conditions (22 ± 3 °C; 50 ± 5% relative humidity) of light/dark cycle of 12 hr with free access to standard food pellets and water *ad libitum*.

#### 2.9.1. Bioavailability study

Animals were randomly divided into three groups (*n* = 3); Group I, received NLC-1; Group II, received AT suspension in 3% carboxymethylcellulose (Choudhary et al., 2012) as a plain drug; and Group III, received commercial tablet (Lipitor^®^ 20 mg). All animals were administrated single oral dose of AT equivalent to 25 mg/kg. At predetermined time intervals, rats were etherized and blood samples were collected from the orbital sinus using a heparinized capillary tube. Samples (1 ml) were collected in heparinized Eppendorf tube and immediately centrifuged at 10,000 rpm for 10 min (Heraeus Biofuge Primo, Thermo Scientific, Waltham, MA). Plasma samples were collected and stored at −20 °C until further analysis using a validated HPLC method.

#### 2.9.2. Chromatography

Plasma samples (200 μl) were transferred to individual Eppendorf tubes containing 200 μl of 10% perchloric acid and vortexed for 30 s at room temperature. Then 500 μl of diethyl ether (extraction solvent) was added and the tubes were centrifuged at 4000 rpm for 15 min (Heraeus Biofuge Primo, Thermo Scientific, Waltham, MA). The supernatant was separated, dried at 40 °C under nitrogen and analyzed. Chromatographic analysis was carried out using the stationary phase, C-18 reverse-phase column (Phenomenex Gemini C18, 250 x 4.6 mm i.d., 5 μ). Mobile phase consisted of filtered and degassed mixture of acetonitrile: 25 mM potassium dihydrogen orthophosphate (50:50, v/v), adjusted to pH 6.5 and was pumped at flow rate 1.0 ml/min. The eluent was detected using UV detector at 246 nm.

#### 2.9.3. Pharmacokinetic parameters

Calculation of maximum concentration (*C*_max_) and time to reach maximum concentration (*T*_max_) were directly obtained from the concentration–time curve. Other pharmacokinetic parameters such as AUC_0–24_ (μg.hr/ml) and AUC_0–∞_ (μg.hr/ml) were carried out using WinNonlinR^®^ 5.3 software (Pharsight Corporation, Cary, NC).

The relative bioavailability of NLC formulations was calculated using the following formula:

(2) %Fr=AUCt/AUCr x 100,

where Fr was the relative bioavailability, AUC_t_ was the area under the plasma concentration–time curve of test (NLC-1), and AUC_r_ was the area under the plasma concentration–time curve of reference samples (Lipitor^®^ and AT suspension).

#### 2.9.4. Pharmacodynamic studies

Animals were randomly allocated into five groups (*n* = 3): Group I, received normal saline (negative control); Group II, administrated fructose/high-fat diet (HFD) (positive control); Group III, fructose/HFD supplemented group treated orally with atorvastatin suspension in 3% carboxymethylcellulose (25 mg/kg) (Choudhary et al., 2012); Group IV, fructose/HFD supplemented group treated orally with NLC-1 (25 mg/kg); and Group V, fructose/HFD supplemented group treated orally with commercial tablet (Lipitor^®^ 20 mg) (25 mg/kg). Fructose was given as a 10% (w/v) solution in drinking water (Vila et al., 2011). HFD consisted of a standard rat diet supplemented with 0.5% cholesterol (Hussein et al., 2011). Fructose and HFD were given for 2 weeks, after which food and fructose solution were removed. Blood samples (1 ml) were collected from retro-orbital sinus under anesthesia with ether. Serum was separated by centrifugation at 10,000 rpm for 10 minutes and samples were stored at −20 °C until analysis. Serum low-density lipoprotein (LDL), high-density lipoprotein (HDL), triglycerides (TG) and total cholesterol (TC) levels were biochemically assayed using commercially available kits (Diamond, Mannheim, Germany).

#### 2.9.5. Histological analysis

At the end of biochemical analysis, animals were killed by decapitation under isoflurane anesthesia and tissue specimens from livers of rats in different groups were fixed in 10% neutral buffered formalin for 24 h and then washed with distilled water followed by dehydration in alcohol. Samples were cleared in xylene and fixed in paraffin bees wax blocks at 56 °C for another 24 h. Sections of 4 μm thickness from the paraffin blocks were cut by sledge microtome, deparaffinized and stained with haematoxylin/eosin. All samples were investigated for steatosis (fatty change of liver) and damage parameters of liver cells as necrosis, pyknosis and congestion using a binocular microscope (Leica, Wetzlar, Germany).

### 2.10. Statistical analysis

All results were expressed as a mean ± SD. The statistical analysis of obtained results was performed by one-way ANOVA followed by Tukey's HSD test using the StatPlus^®^ software (AnalystSoft Inc., Alexandria, VA). Difference at *p* < .05 was considered statistically significant.

## 3. Results and discussion

### 3.1. Encapsulation efficiency

Different components were selected for fabrication of AT-NLCs according to the solubility study performed *via* test tube method (Elbahwy et al., 2017). Selections were based on the high solubilization extent for AT, confirming solubilization of AT in the developed dispersion, which is very important for the optimum drug loading (Marasini et al., 2012). Results revealed that among different lipids screened, the highest solubility of AT was attained in GMS (485 ± 15 mg/gm), Compritol^®^ 888 ATO (82 ± 5.61 mg/gm) and Gelucire^®^ 43/01(7 ± 1.42 mg/gm) which could be attributed to the self-emulsifying property of GMS (Shete & Patravale, 2013), and low HLB values of Gelucire^®^ 43/01(HLB =1) and Compritol^®^ 888 ATO (HLB =2). The lower the HLB value, the more lipophilic the lipid is; and in turn, the higher the HLB value, the more hydrophilic the lipid is. As a result, lipids with low HLB values are more favorable for solubility of hydrophobic AT than lipids with high HLB values. Among various liquid lipids tested, AT revealed very good solubility in Capryol^®^ PGMC (18.43 ± 2.51 mg/ml). AT also displayed good solubility in Pluronic^®^ F68 (9.57 ± 2.39 mg/ml) and Tween^®^ 80 (35.29 ± 3.87 mg/ml). The EE of AT in the developed nanostructured formulations was found to vary between 76 ± 12.4% and 96.6 ± 7.1% (Table 1). Regarding type of solid lipid, it is clear that there was no significant difference (*p* > .05) in EE between tested formulations. The relatively high and insignificant difference might be attributed to complete solubilization of lipophilic AT in Capryol^®^ PGMC which led to massive crystal order defects that left enough space to entrap drug molecules and subsequently improved drug EE (Souto et al., 2004).

Binary mixture of Pluronic^®^ F68 and Tween^®^ 80 displayed lower entrapment efficiencies as compared to corresponding individual use of hydrophilic surfactants (except NLC-9). This behavior could be attributed to higher solubilizing effect of binary mixture to AT than individual use of surfactant. Higher solubilizing effect contributed to partitioning of AT between the oil phase and aqueous phase. As AT has fairly good solubility in the surfactants and solubilizers used, it was pulled out of the oil phase which subsequently reduce amount of AT entrapped in the NLCs (Joshi & Patravale, 2008). To further confirm precision of the measurements, the encapsulated fraction of AT was measured by dissolving pellets of dispersion of NLCs after centrifugation in a solvent mixture of phosphate buffer pH 6.8 and ethanol (1:1). Results revealed that the sum of encapsulated and unencapsulated AT reached 100% of the nominal dose.

### 3.2. Particle size

An important parameter with respect to NLCs as drug carriers is their particle size. As summarized in Table 1, the mean particle size was in the colloidal nanometer range (< 550 nm) for all the prepared formulations except NLC-7 (865.55 ± 7.4 nm) in which Compritol^®^ 888 ATO and Pluronic^®^ F68 were used as solid lipids and hydrophilic surfactant, respectively. This result is in good accordance with our previous published work of thymoquinone-loaded NLCs. It could be attributed to the incompatibility between Compritol^®^ 888 ATO and Pluronic^®^ F68 which was confirmed through stability studies (Elmowafy et al., 2016).

It was observed that mean particle size of different formulations was markedly affected by type of solid lipid. NLCs prepared using Compritol^®^ 888 ATO produced the largest mean particle size while Gelucire^®^ 43/01 produced the smallest NLCs. This might be due to difference in the melting point of lipids, where Compritol^®^ 888 ATO has melting point of 68–75 °C, GMS of 58–60 °C and Gelucire^®^ 43/01 of 43–46 °C. High melting point of solid lipid led to higher melt viscosity and hence decreased efficiency of homogenization step in reducing particle size. To confirm this conclusion, the viscosity of the lipid melt was measured using a Brookfield viscometer (LV model, Brookfield, WI) where viscosities of Gelucire^®^, GMS and Compritol^®^ 888 ATO were found to be 24 ± 2.2, 35 ± 4.8 and 48 ± 2.6 centipoise, respectively. These findings were in agreement with the results reported by Wong et al. (2016).

Additionally, emulsifying properties of Gelucire^®^ 43/01 and GMS facilitated the emulsification and aided in the formation of NLCs with smaller particle size.

The particle size distribution of NLC-3 and NLC-4 showed very good homogeneity as the PDI values were less than 0.3. Other formulations looked heterogeneous as PDI >0.3 which suggested the instability of these formulations. Typically, a small value of PDI (0.2) designates a homogenous vesicle population, whereas larger PDI (> 0.3) indicates a high heterogeneity in particle size distribution (Zhang et al., 2012).

### 3.3. Zeta potential

The zeta potential values of the different formulations were outlined in Table 1. Zeta potential is a significant tool in predicting stability of NLCs. Zeta potential values of more than +30 mV or less than −30 mV are considered enough for stabilization of NLCs (Jia et al., 2010). Zeta potential values ranged from −23 ± 0.28 to −34 ± 6.15 mV. The negative charge was attributed to the anionic nature of the lipid. Surfactants such as Tween^®^ 80 and Pluronic^®^ F68 improved stability of the developed NLCs through their steric hindrance properties. Therefore, most of the developed NLCs are likely to be stable. Moreover, stability of the developed NLCs during storage was investigated by performing stability experiments.

### 3.4. Morphology

**Figure 1.**
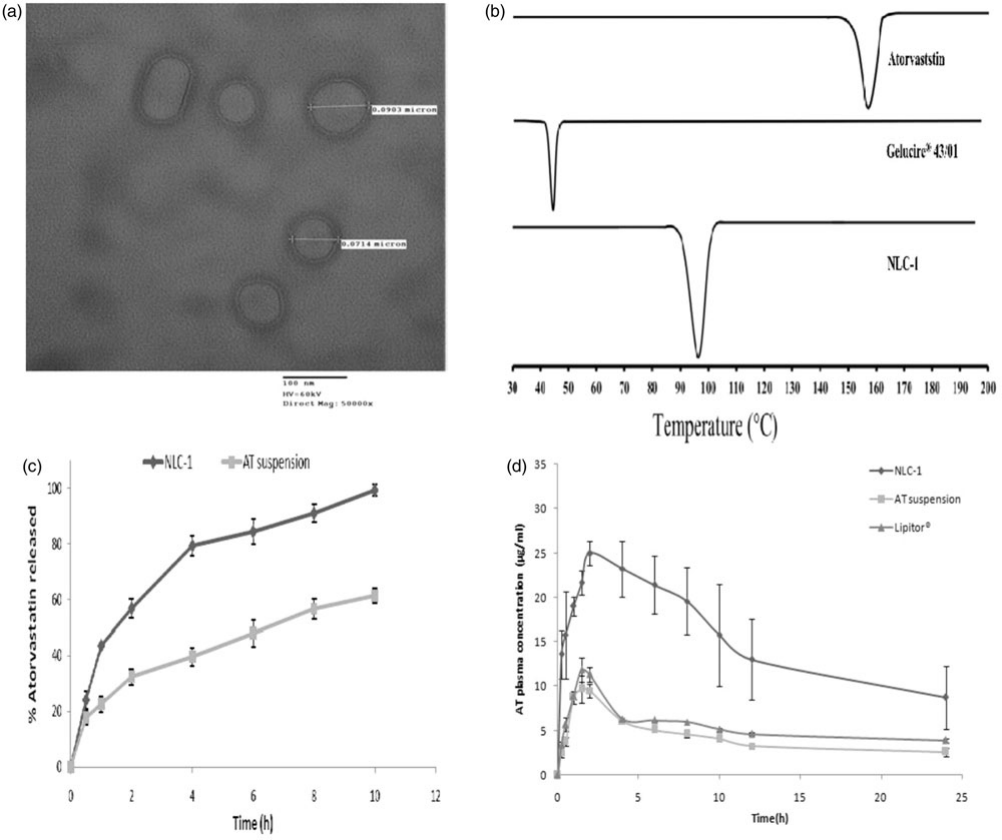
(a) TEM image of optimized NLC-1, (b) DSC thermograms of AT, Gelucire^®^ 43/01and lyophilized NLC-1, (c) *in vitro* release profiles of (▪) AT suspension and (♦) NLC-1 in phosphate buffer pH 6.8, and (d) average plasma concentration vs. time profiles following single oral administration of NLC-1(♦), AT suspension (▪) and (▴) commercial product.

Morphology of the optimized NLC-1 captured by TEM was shown in Figure 1(a). The TEM image revealed that NLCs were of uniform, discrete, and spherical or oval shapes with little or no aggregations indicating that prepared NLCs were homogeneously dispersed. The sizes of NLCs obtained by TEM were in close agreement with the results obtained by a Malvern^®^ Zetasizer (Malvern^®^ Instruments Limited, Worcestershire, UK).

### 3.5. Thermal analysis

DSC is a powerful tool to investigate the crystallization and interaction of drug with different components of NLCs by determining the variation of temperature and energy at phase transition. Figure 1(b) shows DSC thermograms of AT, Gelucire^®^ 43/01and lyophilized powder of developed NLC-1. The thermogram of AT showed a main endothermic peak at around 158 °C representing its melting point and thus indicating crystalline nature. For Gelucire^®^ 43/01, the melting process took place with maximum peaks at 44 °C. Lyophilized NLC-1 showed main endothermic peak at 96.45 °C representing the melting peak of Gelucire^®^ 43/01. The shift in melting peak of Gelucire^®^ 43/01 from 44 °C to 96.45 °C was attributed to the small size of NLCs (nanometer range), dispersed state of the lipid and presence of surfactant. The shift in endothermic peak along with the absence of characteristic AT endothermic peak at 158 °C suggested interaction of AT with lipid component and indicated that AT entrapped in lipids was in amorphous state or dispersed molecularly in the NLCs.

### 3.6. In vitro release studies

The *in vitro* release study was performed using reverse dialysis technique in phosphate buffer pH 6.8. Two reasons make reverse dialysis appropriately correlates with the *in vivo* release behavior than the conventional dialysis. Firstly, as the colloidal system is directly placed into the release medium, maximum drug dilution will occur (perfect sink conditions) and drug will therefore diffuse out from the oily droplets to the sink solution. Drug dissolved in the donor aqueous solution will easily diffuse through membranes of dialysis bags (Levy & Benita, 1990). Secondly, large membrane surface area which is available for drug diffusion as the dialysis bag is in direct contact with the extended sink solution (Levy & Benita, 1990). From experimental point of view, reverse dialysis technique resulted in a significant reduction in the variation of dissolution data as compared to the conventional dialysis membrane method (Xu et al., 2012).

The *in vitro* release profiles of NLC-1 compared to pure AT suspension are displayed in Figure 1(c). NLC-1 formulation was chosen for *in vitro* release study based on the smallest particle size, highest zeta potential and appropriate entrapment efficiency. It is clear that AT released from NLC-1 was nearly complete (99.3 ± 2.17%) while pure AT suspension released about 61.3 ± 2.5% after 10 h. Dissolution enhancement is the primary objective of such drugs specially those categorized as class II, rate limiting step in drug absorption. Moreover, NLC-1 showed biphasic release behavior; the first phase lasted 4 h with relatively rapid release which may be due to AT enriched in the shell around the particles along with the steric stabilization effect of Pluronic^®^ F68 which covered surface of NLCs that can entrap some AT molecules. This stage was followed by slower release phase lasting up to 10 h as a result of slow diffusion of drug from lipid matrix (Ravi et al., 2014).

By applying kinetic fit, AT release from NLC-1 in pH 6.8 was most appropriately fitted to Higuchi diffusion model (*r* = 0.9829) indicating lipid matrix erosion. By comparison with other models, zero-order kinetics (*r* = 0.9053), first-order kinetics (*r* = 0.4605) and Hixson–Crowell model (*r* = 0.9803) exhibited relatively smaller *r* values. Linear regression of NLC-1 that the time required for 50% AT release (t_1/2_) was calculated as 2.5 h while the release rate constant was about 31 h^−1^.

### 3.7. Stability of NLCs

Stability study was performed on the basis of particle size, PDI, zeta potential and entrapment efficiency during three months at 2–8 and 25 °C (Table 2). The results obtained by end of the three months of study revealed that NLCs stored at both temperatures remained in the colloidal nanometer range (<550 nm). NLCs stored at 25 °C displayed a trend of extensive particles growth, increase in PDI, and decrease in zeta potential and EE. This may be attributed to polymorphic behavior of the solid lipid incorporated in formulation of NLCs which undergo transformation to the more stable β-polymorph upon exposure to kinetic energy (light or temp). NLCs stored at 2–8 °C revealed no pronounced changes in the measured parameters which suggest 2–8 °C as an optimum storage temperature for NLCs. These results are in agreement with Sanad et al. who studied the effect of temperature on stability of oxybenzone-loaded NLC (Sanad et al., 2010).

**Table 2.** Stability study of NLC-1.

Storage condition	Mean particle size ± SD (nm)	PDI	Zeta potential ± SD (mV)	Entrapment efficiency ± SD (%)
Fresh	162.5 ± 12	0.295	−34 ± 0.29	90.1 ± 6.5
2–8 °C				
1 month	173 ± 4.21	0.298	−32.4 ± 0.82	89.45 ± 1.4
3 months	191 ± 3.67	0.308	−32.4 ± 1.37	88.18 ± 1.3
25 °C				
1 month	198.6 ± 4.61	0.341	−28.7 ± 0.65	86.12 ± 0.73
3 months	273.6 ± 6.8	0.393	−22.1 ± 0.79	83.31 ± 0.82

### 3.8. Bioavailability study

In order to explore possibility of utilizing NLCs to improve oral bioavailability of AT, pharmacokinetic study was performed and pharmacokinetic parameters were calculated for NLC-1, AT suspension and commercial product (Lipitor^®^). The mean AT plasma concentration–time profiles are shown in Figure 1(d) and the calculated pharmacokinetic parameters are tabulated in Table 3. NLC-1 differed significantly (*p* < .05) in their pharmacokinetic parameters from AT suspension and commercial product. The *T*_max_ of AT suspension and Lipitor^®^ was 1.5 h while the *C*_max_ values were 9.7 ± 1.5 μg/ml and 11.9 ± 1.3 μg/ml, respectively. However, the time required to reach maximum concentration was delayed by 0.5 h for NLC-1. The *C*_max_ of NLC-1 (25 ± 1.4 μg/ml) was significantly (*p* < .05) higher than both AT suspension and Lipitor^®^ which might be attributed to the reduction in particle size from micron (AT suspension and commercial) to nanometer range (NLC-1) which consequently increased the effective surface area and hence the solubility and dissolution rate and extent of AT, the rate determining step in the absorption (Hörter & Dressman, 2001). Moreover, the nanosized particles play a crucial role in certain intestinal absorption mechanisms as paracellular transport, transcytosis and/or uptake by M-cells (He et al., 2012).

**Table 3.** Pharmacokinetic data of AT following single oral administration (25 mg/kg) of NLC-1, AT suspension and commercial tablet to rats.

Pharmacokinetic parameters	NLC-1	AT suspension	Lipitor^®^
*C*_max_ (μg/ml)	25 ± 1.4^a^	9.7 ± 1.5	11.9 ± 1.3
*T*_max_ (hr)	2 ± 0.25^a^	1.5 ± 0.15	1.5
AUC_0–∞_ (μg^a^hr/ml)	599 ± 224.3^a^	168.1 ± 27	286.3 ± 38.5
AUC_0–24_ (μg^a^hr/ml)	370.9 ± 65^a^	101.8 ± 2.7	129.3 ± 3.7
Relative bioavailability		3.56	2.09

Each value expressed as the mean ± SD (*n* = 3).

^a^ Significant at *p* < .05.

The extent of AT absorption from NLC-1, represented in AUC_0–24_ and AUC_0–end_, was significantly higher (*p* < .05) than AT suspension and Lipitor^®^. Relative bioavailability of NLC-1 was ∼3.6- and 2.1-fold higher than AT suspension and Lipitor^®^, respectively, indicating significant enhancement of oral delivery of AT when incorporating in NLCs. A recent study has compared bioavailability of freeze-dried atorvastatin calcium-loaded poly-ɛ-caprolactone nanoparticles with Lipitor^®^ and reported lower relative bioavailability of nanoparticles than Lipitor^®^ at 10 mg/kg dose and equal efficacy at lower doses. The authors suggested that AT calcium in nanoparticles might be mainly distributed into the liver rather than in the blood (Ahmed et al., 2016). The supposed analysis confirmed higher fraction of unencapsulated drug in polymeric nanoparticles (nanospheres; as the authors did not use oil) which in turn absorbed by mesenteric pathway. The superiority of NLCs over polymeric nanospheres in oral bioavailability enhancement lies in two factors: firstly, lipid contents of NLCs which resemble fatty meal that stimulate bile secretion into small intestine which associate with NLCs to form mixed micelles (Chen et al., 2010; Zhuang et al., 2010; Tiwari & Pathak, 2011); and secondly, higher encapsulation efficiency as NLCs containing liquid lipid which easily dissolve the drug. Collectively, both factors might help lymphatic absorption and avoidance of first-pass effect especially for sparingly soluble drugs. Moreover, NLC-1 is considered as promising carrier for enhancing oral bioavailability of AT as its components are reported to correct bioavailability drawbacks of AT. Gelucire^®^ 43/01 was reported to reduce the active secretion by the efflux transporter (Pgp) due to modifying protein expression within Caco-2 cells (Sachs-Barrable et al., 2007). Poloxamer block co-polymers showed steric stabilization on the lipid and hence little degradation activity of lipase was observed in turbidity testing (Müller et al., 1996). It also reduces affinity and/or accessibility for the substrate-binding sites of the cells overexpressing Pgp and MRPs efflux proteins (Batrakova et al., 2004). Additionally, NLCs are stable under simulated stomach condition after 2-h digestion in terms of particle size and PDI (Aditya et al., 2014), reported to be partly responsible for variable bioavailability of AT (Shah et al., 2008). The presence of lecithin as lipophilic surfactant might influence the uptake of NLCs by Peyer’s patches. These results are supported by Cavalli et al. (2003).

### 3.9. Pharmacodynamic studies

Pharmacodynamic studies focused on measurement of serum lipids and histological finding of liver tissue as they are considered as the major pharmacological effects of AT.

#### 3.9.1. Serum lipid analysis

HFD is well recognized as a predisposing factor in the development of hypercholesterolemia and fructose administration in drinking water (10% w/v) for 14 days induced hypertriglyceridemia and hepatic steatosis (Vila et al., 2008). Serum lipid profiles are shown in Table 4. It is obvious that the serum TC was significantly (*p* < .05) increased in Group II to an average of 98.5 ± 2.67 mg/dl compared with Group I average of 50.8 ± 6.87 mg/dl indicating occurrence of hypercholesterolemia with HFD. AT-treated groups (groups III, IV and V; treated with AT suspension, NLC-1 and Lipitor^®^, respectively) showed marked decrease in TC levels (59.45 ± 4.34, 45.94 ± 3.76 and 54.7 ± 6.17 mg/dl, respectively) when compared with Group II while no significant difference detected among AT-treated groups (*p* > .05). Results of TG and LDL levels also showed significant increase in Group II (178 ± 9.14 and 86.8 ± 6.48 mg/dl, respectively) when compared with Group I (73 ± 5.24 and 12.87 ± 8.64 mg/dl, respectively). By AT treatment, significant decrease in TG and LDL levels was observed. Concerning HDL values, the highest elevation was observed in Group IV (NLC-1 treated group; 37.84 ± 1.79 mg/dl), reflecting superiority of the NLCs formulation. It is well-known that HDL has a protective role in CVD as it enhances cholesterol removal from peripheral tissues to the liver for catabolism and excretion (Chen & Pan, 2007).

**Table 4.** Effect of NLC-1, ATC suspension and Lipitor^®^ tablet on serum level of total cholesterol, triglyceride, high-density lipoprotein and low-density lipoprotein.

Group	TC (mg/dl)	TG(mg/dl)	LDL(mg/dl)	HDL (mg/dl)
I (negative control)	50.8 ± 6.87	73 ± 5.24	12.87 ± 8.64	33.26 ± 6.93
II (positive control)	98.5 ± 2.67	178 ± 9.14	86.8 ± 6.48	29.09 ± 5.98
III (atorvastatin suspension)	59.45 ± 4.34^a^	123.73 ± 5.11^a^	48.37 ± 6.4^a^	31.39 ± 2.95
IV (NLC-1)	45.94 ± 3.76^a^	99 ± 4.67^a^	23.16 ± 3.97^a^	37.84 ± 1.79
V (Lipitor^®^)	54.7 ± 6.17^a^	115.85 ± 7.49^a^	38.63 ± 9.43^a^	33.34 ± 4.68

Each value expressed as the mean ± SD (*n* = 3).

^a^ Significant at *p* < .05 as compared to positive control group.

#### 3.9.2. Histological examination

**Figure 2.**
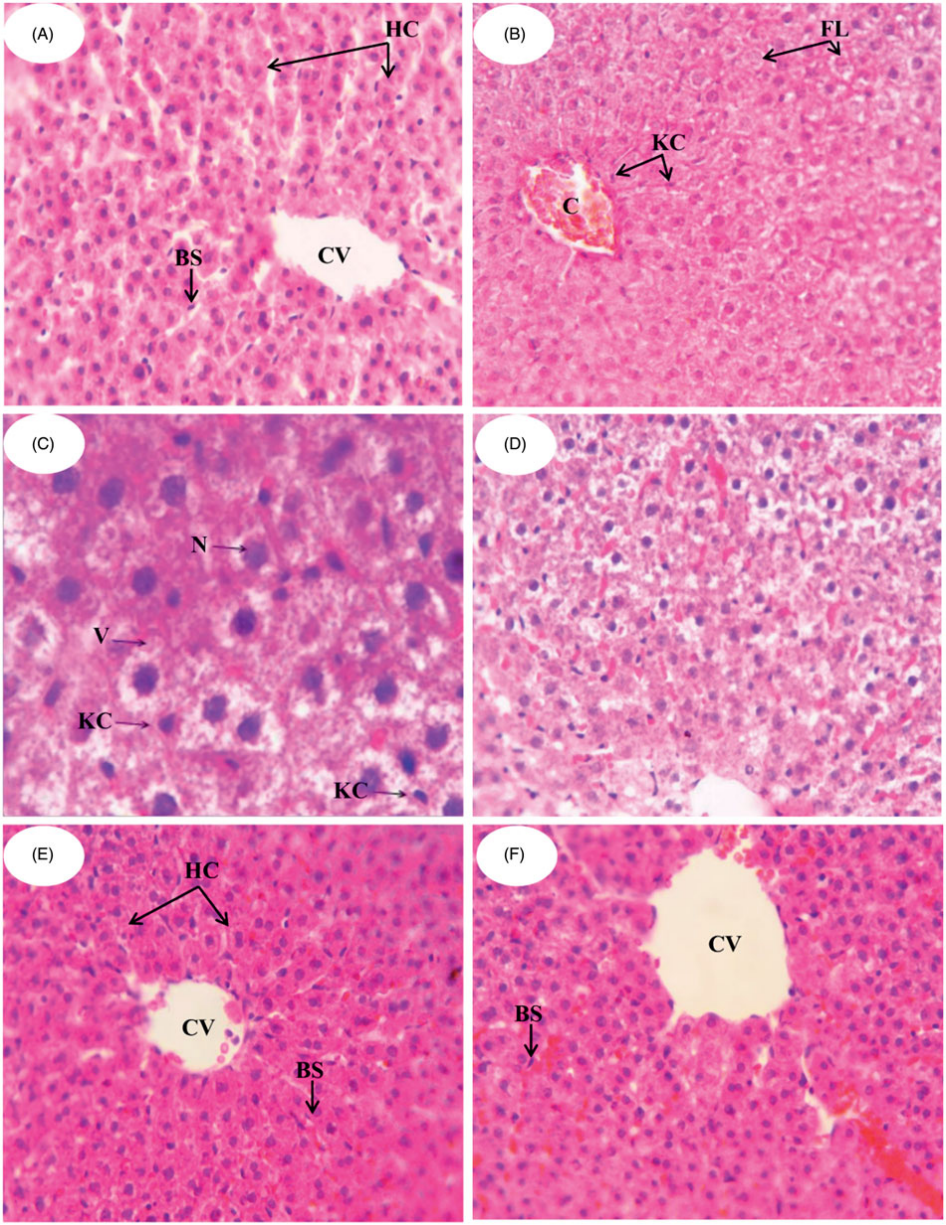
Photomicrographs of the liver sections in male albino rats (H&E) (A) control group, (B,C) group fed fructose/HFD supplements, (D) group treated with AT suspension, (E) group treated with NLC-1 and (F) group treated with commercial product.

Liver biopsy and histological examination remain the most beneficial approach for appropriate evaluation of anti-hyperlipidemic activity of the developed formulations (Kleiner et al., 2005). Figure 2 shows the photomicrographs of histological sections of liver tissues from different groups. Figure 2(A) shows the control group (Group I) in which liver section exhibits regular liver architecture with normal polygonal hepatic cells (HC) with round nuclei, normal central vein (CV) and blood sinusoids (BS). On the contrary, Group II (Figure 2 (B,C)) shows abnormal architectural organization of hepatic tissue, dilatation and congestion in central vein (CV). Hepatocytes were manifested by marked cytoplasmic vacuolation (V), focal necrosis (N) and fatty change liver (FC) with diffused Kupffer cell (KC) proliferation in between. It was reported that HFD and thus hypercholesterolemia are considered as a risk factor for hepatic fibrosis as well as atherosclerosis and coronary artery disease (Jeong et al., 2005). Also, high fructose intake in rats induced hepatic steatosis and a clear state of liver inflammation (Vila et al., 2011). Administration of AT suspension (Group III; Figure 2(D)) shows a mild improvement in liver cells structure but there was a fatty change liver, and many hepatocytes showed ballooning and contain Mallory’s hyaline. There was a mixed infiltrate of inflammatory cells including a prominent component of neutrophils. In Figure 2(E), Group IV administered with NLC-1 showed improvements of hepatocyte structures. Restoration of normal histological structures of liver represented in hepatic strands, Kupffer cells (KCs) and central vein (CV) was attained. It is clear that NLC-1 remarkably reduced the histological changes when compared to that observed in groups II and III. It is worth mentioning that AT can improve nonalcoholic steatohepatitis (NASH), a progressive form of NAFLD that may proceed to end-stage liver diseases (Zheng et al., 2008), by decreasing tumor necrosis factor-α (TNF-α) levels (Hyogo et al., 2012). AT also prevents hepatic carbohydrate response element binding activation by activating protein kinase A, thus providing a plausible molecular mechanism for the therapeutic effect of atorvastatin on NAFLD (Vila et al., 2011). By comparison, Lipitor^®^ administered group (Group V; Figure 2(F)) showed less hepatic improvements than NLC-1 administered group as the former exhibited a mixed infiltrate of inflammatory cells, including a prominent component of neutrophils. Results of histological studies are in good accordance with that of serum lipid profiles of different groups confirming the superiority of NLC-1 as compared to other groups.

## 4. Conclusions

AT-loaded NLCs were successfully prepared using different solid lipids and surfactants. The formulations were characterized and showed particle size ranging between 162.5 ± 12 and 865.55 ± 28 nm, zeta potential values ranging between −34 ± 0.29 and −23 ± 0.36 mV, and encapsulation efficiencies higher than 87%. DSC study gave evidence for existence of AT in an amorphous state with improved dissolution rate and extent. The optimized formulation (NLC-1) showed higher release than corresponding suspension. Oral bioavailability of NLC-1 increased over 3.5- and 2-fold when compared with AT suspension and Lipitor^®^, respectively. Higher plasma concentration of AT from NLCs was attributed to nanosize and formulation excipients that might augment lymphatic absorption. Serum lipid profiles demonstrated that NLC-1 has the potential to lower hyperlipidemia. Furthermore, treatment with NLC-1 could meaningfully restore the liver tissue histology after intoxication with cholesterol and fructose. In conclusion, NLCs of the poorly water-soluble AT was an effective approach for improving its oral bioavailability and pharmacological bioactivity.
